# Current context and the experience of Brazilian Medical Societies with Specialist Title Exams

**DOI:** 10.1590/0100-6991e-20243863EDIT01-en

**Published:** 2024-12-12

**Authors:** GERSON ALVES PEREIRA, RAMIRO COLLEONI, JAIR GIAMPANI, JORGE CARVALHO GUEDE, ANA CRISTINA KFOURI CAMARGO, FÁBIO TERUO MATSUNAGA, ROSELI MIEKO YAMAMOTO NOMURA, FELIPE OLIVEIRA MARQUES, JOSÉ EDUARDO LUTAIF, CESAR EDUARDO FERNANDES

**Affiliations:** 1 - Universidade de São Paulo, Campus Bauru, SP, Brasil. Presidente da Comissão do Título de Especialista do Colégio Brasileiro de Cirurgiões; 2 - Universidade Federal de São Paulo, SP, Brasil. Presidente da Comissão de Residência Médica do Colégio Brasileiro de Cirurgiões; 3 - Universidade Federal de Mato Grosso, Cuiabá, Brasil. Coordenador da Comissão de Ensino do Conselho Brasileiro de Oftalmologia; 4 - Universidade Federal da Bahia, Coordenador da Comissão do Título de Especialista da Federação Brasileira de Gastroenterologia; 5 - Faculdade de Ciências Médicas da Santa Casa de São Paulo, Brasil. Colaboradora Eleita do Título de Especialista em Otorrinolaringologia pela ABORL-CCF; 6 - Universidade Federal de São Paulo, SP, Brasil. Comissão de Ensino e Treinamento da Sociedade Brasileira de Ortopedia e Traumatologia (SBOT); 7 - Universidade Federal de São Paulo, SP, Brasil. Comissão Nacional do TEGO - Título de Especialista em Ginecologia e Obstetrícia; 8 - Presidente da Comissão de Certificação em Anestesiologia da SBA; 9 - Faculdade de Ciências Médicas da Santa Casa de São Paulo, Brasil. Diretor Científico da Associação Médica Brasileira (AMB); 10 - Faculdade de Medicina do ABC, Santo André, SP, Brasil. Presidente da Associação Médica Brasileira (AMB)

**Keywords:** Medical Societies, Professional Training, Health Care Quality, Academic Performance, Sociedades Médicas, Capacitação Profissional, Garantia da Qualidade dos Cuidados de Saúde, Desempenho Acadêmico

## Abstract

The editorial discusses the need for the Specialist Title to be valued by Medical Societies, as a stage after the conclusion of medical residency and a mandatory prerequisite for taking the tests. Finally, it shows the experience of seven Medical Societies with their specialist title exams.

## RESUMO

O editorial discute a necessidade de valorização do Título de Especialista pelas Sociedades Médicas, como etapa posterior à conclusão da residência médica e pré-requisito obrigatório para prestar as provas. Por fim, mostra a experiência de sete Sociedades Médicas para suas provas de título de especialista.

## INTRODUCTION

For any of the 55 specialties and 62 areas of medical practice recognized in Brazil (Brazil, 2024),, the Federal Council of Medicine, through its Regional Councils (CRM), can only register as specialists (granting the Specialist Qualification Registration - RQE) physicians who present: 1) a Certificate of Completion of Medical Residency accredited by the National Commission of Medical Residency (CNRM) and/or 2) a Specialist Title (ST) granted by a Brazilian Association or Society of the respective specialty affiliated to the Brazilian Medical Association (AMB) and whose public notice for the Specialist Title exam followed the rules of the AMB and is approved by it (FALK, 2006; AMB, 2021).

Medical Residency and Specialist Title are certificates of different nature, being independent. A doctor may have one or both, but each entitles the specialist to register as such with the CRM. By determination of the AMB, a Specialist Title is no longer to be granted only due to excellent curriculum or proof of completion of Medical Residency (FALK, 2006). Currently, it is necessary to pass exams that involve a written test (cognitive assessment) and a practical one (assessment of technical and communication skills).

Unlike what the legislation allows in Brazil, where one can obtain the RQE issued by the CRM only with the medical residency program (MRP) completion certificate, other countries have a very different policy from ours for issuing ST. Medical residency is a necessary and indispensable prerequisite for the candidate to register and take the ST exams. Without medical residency, application for the exams is not even allowed. These are conducted through cognitive and practical evaluations by a panel of independent experts for the effective proof of competencies, skills, and attitudes, after which only then the ST is granted to the approved candidate. Never, however, do they admit a ST issuance based exclusively on the medical residency completion certificate. This is indeed a necessary condition, but by no means sufficient for the ST granting (Fernandes, 2024).

This double path to titling must have a deadline to end (Oliveira, 2024). Titles by scientific societies should be granted, with strict criteria, only to specialist physicians trained through residency programs formally accredited by the National Commission for Medical Residency (CNRM). On the one hand, it is evident that for this to occur, there is a concomitant imperative to expand the quantity and quality of residency programs in all specialties (and according to the needs of the country) and ensure adequate amounts of funds for the residency scholarship (Oliveira, 2024).

In their early years, Medical Societies allowed the enrollment of physicians without residency to take the ST exam. Of course, the intention at that time was, on a provisional basis, to give the opportunity for many seasoned doctors with no residency but with solid proven experience to have the opportunity to render their Specialist Title official if approved in the exams. This was justified since MRPs were scarce and only a small portion of medical school graduates sought this type of training. This situation without any standardization lasted for a long time, and the regulation of medical residency only occurred in 1977 through a presidential decree defining that the MRP would constitute a modality of postgraduate education for physicians, in the form of a specialization course, characterized by in-service training, in a regime of exclusive dedication, functioning in health institutions, university or not, under the guidance of medical professionals with high ethical and professional qualifications (Brasil, 1977). It would not be advisable to prevent all other doctors who did not meet these prerequisites from taking the test, at that time, 47 years ago. It is clear, therefore, that this initial permission for doctors without medical residency to take the ST exams should be understood as transition rules, to be extinguished as soon as possible. Nowadays, that idea of allowing doctors without residency to take the ST exam no longer has any reason to exist because the doctors from that time are no longer active. But the fact is, this permission still exists. This situation can only change by legal provision. Many decades have passed, and now we have MRP graduates in large numbers, supposedly formed with the competence matrices required for the adequate training of the medical specialist. We agree this needs to change. We need to address this issue with the same responsibility verified in countries that treat the training of medical specialists with the necessary seriousness. Also, we need to institute, here in Brazil, the completion of a MRP as a mandatory prerequisite to the ST exam application. It is past time we apply seriousness to our criteria for granting the ST (Fernandes, 2024). 

This enduring distortion is exemplified by category E of the Brazilian College of Surgeons’ Specialist Title Exam, where candidates must prove six years of experience as a general surgeon in Brazil through a statement and list of surgeries performed in the last 24 months up to the time of registration, signed by the Hospital Director and/or Chief of the Surgery Service. Proof can be provided through documents issued by several Services and Hospitals that together add up to the minimum six required years. The surgical procedures, for which there must be mandatory proof of performance as the “Responsible Surgeon”, must be both elective and emergency procedures provided for in the competency and content matrix of General Surgery at the National Medical Residency Commission (CNCRM).

The Specialist Titles awarded by Medical Societies serve as an indicator of competence, quality, and professional ethics in medical practice. By establishing strict criteria for certification, requiring continuous professional updating, and monitoring the practice of holders, Medical Societies play a key role in protecting against professional malpractice and promoting high health care standards (Pereira Júnior et al, 2024).

It is very interesting the wealth of stories and experiences of each of the Medical Societies in the construction of their specialist title tests in relation to: 1) Year of the Society creation; 2) Year of specialist title exam start; 3) types of assessment (cognitive - multiple-choice and written tests; oral - discussion of clinical cases; face-to-face or online simulated stations; and other types of assessment); 4) how to prepare each exam; 5) approval criteria; 6) form of evaluators selection and training; 7) form of results publication; 8) way of approaching appeals and questions about the various types of exams; 9) offer of a training course for candidates (if so, what type); and 10) title validity and recognition time.

The table below compares the main information and indicators about the specialist title exam of seven of the most traditional Medical Societies..

[Table t1]

**Table t1:** 

INDICATORS	ABORL/CCF	SBA	FEBRASGO	CBO	SBOT	FBG	CBC
Year of the Society's creation	November 21, 1978	1948	October 30, 1959	1941	1935	1949	1929
Starting year of the specialist title exam	XX Brazilian Congress of ENT, in 1971.	1983	1966	1960	1972	1957	1971
Current evaluation phases	Theoretical and Theoretical-practical; curriculum vitae	Two phases	Online Theoretical/Face-to-face Practice	Online test and practical test	Two phases	Single phase	Two phases (Online test and practical test)
Types of evaluation (starting year and number of questions, cases, and stations of each): - Cognitive (multiple-choice, written tests) - Oral (discussion of clinical cases) - Simulated stations - Other types of assessment	- Theoretical: 80 multiple-choice questions (weight=4.5) - Curriculum vitae analysis (weight=1) - Theoretical-practical: clinical cases of six subareas of Otorhinolaryngology (sleep, rhinology, pharyngostomatology, laryngology, otology, and otoneurology) (weight=4.5)	First phase objective test with 80 questions Second phase with 10 clinical cases, written. No oral evaluation at the moment	- Cognitive test by Multiple Choice Tests. 120 questions, 60 gynecology and 60 obstetrics - Practical test with 8 simulated practical cases (4 gynecology cases and 4 obstetrics cases)	- Basic Exam: 50 questions multiple choice online; - Clinical Surgical Test: 100 online multiple-choice questions; - Practical Theoretical Test: 50 online multiple choice questions; - Practical test in specific locations; - No oral evaluation at the moment.	Sets of Tests: - Theoretical: 120 online multiple-choice questions; - Theoretical-practical: 16 clinical cases, oral in person with two examiners; -Practice: - Physical examination: five situations with one hired mannequin and two examiners; - Attitudes: one situation (along with physical examination) - Skills: five situations with two examiners - Scientific work: scientific work required for registration in TEOT.	2020- Multiple choice test with 100 items (70%) + curriculum analysis with barema (30%)	First phase - elimination - Theoretical test - 100 multiple-choice questions Second phase - Oral practical test with discussion of clinical cases (since 1988). - Simulated practical test in person and online (since 2022)
Exam preparation	Face-to-face meeting of the members of the Commission	SBA-designated committee	Specific commission	Professional Exam Commission	eaching and Training Commission (CET)	Based on a thematic and competence matrix, questions prepared by the Title Committee.	Specialist Title Commission (COTECIG)
INDICATORS	ABORL/CCF	SBA	FEBRASGO	CBO	SBOT	FBG	CBC
Approval Criteria	Minimum score of 6 (six) in the theoretical test (T) and in the theoretical-practical test (TP) and final weight average of 6 (six) as well. Weights: T=4.5; TP=4.5; Curriculum Vitae=1	Minimum success rate of 60% in both phases.	Theoretical phase equal to or greater than 7.00 and practical equal to or greater than 6.50; for approval, sum of theoretical and practical with an average equal to or greater than 7.00	Online exams: arithmetic average equal to or greater than 6.5, with a minimum grade of 6.0 in each of the exams. Practical test grade equal to or greater than 7.0	Minimum score for each set of tests (theoretical; theoretical-practical and practical) 50%. The scientific work has no minimum score, but is required for registration Minimum total score: 60% The following weights are: • Online test: 40%; • Oral test: 28%; • Physical exam and behavior test: 14%; • Skills test: 14%; and • Scientific work: 4%.	Minimum score of 7.0 (seven) in the weighted sum of the evaluations	Candidates who obtain at least 70% of the highest score and who have an overall score of at least 50% of the highest score will pass the Written Test. To pass the second phase, candidates must have an average of 7.0 (seven) in the oral and simulated tests. They must also obtain at least 50% of the maximum score in each set of practical tests (Discussion of Clinical Cases in the oral test and Practical Stations in the simulated test, in person or online) to avoid failing.
Selection and training of Evaluators	Otorhinolaryngologists who are members of ABORL-CCF and who have had the Specialist Title for at least 3 (three) years.	Holders of the SBA Higher Degree in Anesthesiology and election at a specific annual meeting.	TEGO Graduates	Outsourced company	The TEOT Examiner must be appointed by an accredited service, have at least 5 years passed the TEOT and be in good standing with the SBOT	Nomination by the Presidency among the professors with experience in medical education.	Online training through instructional videos for the evaluators of the clinical cases or simulated stations they will examine.
INDICATORS	ABORL/CCF	SBA	FEBRASGO	CBO	SBOT	FBG	CBC
Publication of results	Through the system exposed in the Notice (ABORL-CCF website)	SBA website.	FEBRASGO website about 40 days after the practical test	Online platform	Approved List: Website Notes, individually on the portal	Individual Letters of Approval	Individual consultation on the websites of the applying companies.
Way of approaching the appeals and questions about the various exams	Appeals are filed online and answered individually, explaining, when rejected, why, both the questions of the theoretical test and the theoretical-practical test!	Website of the company hired to apply the test (eduCat).	Appeals are sent by a specific platform, on the candidate’s page and analyzed by the title committee	Follow the rules of the respective notices	Cognitive theoretical test and scientific work: online resource being analyzed by CET. Other tests: scheduling for verification of the test book and the score given by the examiners of each question, in the SBOT.	Analysis of all appeals with justifications. Legal analysis, if necessary, with own legal advice. Referral to AMB if necessary.	Follow the rules of the respective notices
Is there a training course offered for candidates (if yes, what type)	It is known today that there are several preparatory “courses” for the INDEPENDENT Specialist Title Test of the Specialist Title Committee and ABORL-CCF.	Yes. Classes available on the SBA website for members.	There are preparatory courses offered by private companies	Yes, online course	SBOT and especially the Regional Offices offer preparatory courses for the title test with simulations for the theoretical, theoretical-practical, and practical tests.	Online courses throughout the year (Gastroenterology Course, Young Gastro and FAPEGE) in addition to the “FBG University” with material available Online in repository	No
Validity Time and Recognition of the Title	Indefinite	Lifetime title	The title has no expiration date, recognition of the veracity of the title is checked with the existing documentation and verification of the number and year of the exam	Indefinite	Indefinite	Lifelong	Indefinite
Revalidation/recertification of the title	No need for revalidation	None	None	None	None	Not applicable	None

*SBA - Sociedade Brasileira de Anestesiologia; SBOT - Sociedade Brasileira de Ortopedia e Traumatologia; CBO - Conselho Brasileiro de Oftalmologia; ABORL-CCF - Associação Brasileira de Otorrinolaringologia e Cirurgia Craniofacial; FBG - Federação Brasileira de Gastroenterologia; CBC - Colégio Brasileiro de Cirurgiões; FEBRASGO - Federação Brasileira das Associações de Ginecologia e Obstetrícia.*
